# Body composition in stable COPD: associations with disease severity and early sarcopenia prediction

**DOI:** 10.3389/fnut.2026.1862289

**Published:** 2026-09-01

**Authors:** Huifang Zhang, Fenkai Guo, Juanxia Chen, Huijuan Ma, Xiuhe Kang, Yuanyuan Yang, Lijun Chen

**Affiliations:** 1 Department of Respiratory and Critical Care Medicine, The First People’s Hospital of Yinchuan, The Second Clinical Medical College of Ningxia Medical University, Yinchuan, China; 2 Department of Emergency Medicine, General Hospital of Ningxia Medical University, Yinchuan, China

**Keywords:** bioelectrical impedance analysis, body composition analysis, chronic obstructive pulmonary disease, sarcopenia, skeletal muscle index

## Abstract

**Objective:**

To investigate body composition changes in patients with stable chronic obstructive pulmonary disease (COPD) and their correlation with disease severity, and to assess the value of body composition indices for early identification of concomitant sarcopenia.

**Methods:**

Patients with stable COPD were stratified according to the comprehensive disease severity assessment method in the Global Initiative for Chronic Obstructive Lung Disease (GOLD) guidelines, changes in body mass index (BMI), skeletal muscle index (SMI), body fat mass index (BFMI), and fat-free mass index (FFMI) were observed across groups. Factors associated with concomitant sarcopenia in COPD were analyzed, and Receiver Operating Characteristic (ROC) curves were plotted to evaluate the predictive value of the body composition indices for early screening of concomitant sarcopenia in patients with stable COPD.

**Results:**

A total of 237 patients with stable COPD (including 40 with concomitant sarcopenia) and 270 healthy controls were enrolled. In the stable COPD group, BMI, SMI, and FFMI were all positively correlated with FEV₁%pred and FEV₁/FVC (all *p* < 0.05), with correlation coefficients ranging from 0.142 to 0.194, and negatively correlated with modified Medical Research Council (mMRC) dyspnea score, COPD Assessment Test (CAT) score, and number of acute exacerbations in the past 1 year (*r* = −0.405, −0.405, −0.174, *p* < 0.01; *r* = −0.336, −0.298, −0.208, *p* < 0.01). SMI and FEV₁%pred were independent predictors of concomitant sarcopenia (OR = 0.011, 0.868; *p* < 0.05). The combined SMI and FEV₁%pred showed superior diagnostic performance (AUC = 0.990, 95%CI: 0.981–0.998).

**Conclusion:**

Body composition alterations occur during the stable phase of COPD, especially a reduction in skeletal muscle mass. Changes in skeletal muscle indices in patients with stable COPD are significantly correlated with disease severity. Body composition analysis has potential clinical value for early identification of skeletal muscle impairment and disease assessment in stable COPD patients, and may be considered as a supplementary component of routine follow-up.

## 1 Introduction

Chronic obstructive pulmonary disease (COPD) is a prevalent, complex respiratory disorder characterized by multisystem involvement. The condition typically demonstrates insidious onset and is associated with diverse risk factors, contributing to its recognition as a ‘silent killer’ (1). COPD imposes a substantial health burden in China, characterized by persistently elevated prevalence and mortality rates. It has been designated as one of the four primary chronic diseases to be addressed in the national health plan, ‘Health China 2030’ (2–4). COPD presents dual clinical challenges that synergistically accelerate functional decline: substantial diagnostic delays averaging >5 years in primary care settings and frequent acute exacerbations (≥2 events/year in 40% of advanced cases) (5). These factors collectively drive pulmonary deterioration at approximately twice the rate observed in healthy aging (mean FEV1 decline: 60–80 mL/year vs. 30 mL/year) (6). This respiratory compromise induces systemic musculoskeletal degeneration through hypoxia-mediated molecular pathways, including ubiquitin-proteasome system activation and myostatin upregulation. The resultant sarcopenic phenotype, defined by appendicular skeletal muscle index <7.0 kg/m^2^ in males and <5.7 kg/m^2^ in females, establishes a self-perpetuating cycle that amplifies dyspnea through impaired ventilatory mechanics while elevating 5-year mortality risk by 3.2-fold compared to COPD patients with preserved muscle mass (7, 8).

Currently, concomitant sarcopenia in COPD has become a major clinical concern. In 2014, the European Respiratory Society (9) proposed a method for assessing the nutritional status of COPD patients based on anthropometric measurements. However, multinational registry data (2020–2023) demonstrate alarmingly low clinical adoption rates, with only 15.8% of GOLD B-D patients undergoing multifrequency bioimpedance analysis during pulmonary rehabilitation enrollment (10). In addition, few studies have been reported on the correlation between body composition indices and multidimensional disease assessment indicators in COPD patients. Furthermore, most existing studies have focused on patients with acute exacerbations or advanced COPD (11), while evidence on body composition changes in the stable phase and their associations with pulmonary function, symptom burden, and exacerbation risk remains limited. There is also a lack of clinically practical screening tools for early identification of sarcopenia in stable COPD populations (12), which hinders timely nutritional and rehabilitative interventions.

In this study, we measured the body composition in stable COPD patients and healthy physical examination participants using a body composition analyzer. Key parameters included body mass index (BMI), skeletal muscle index (SMI), body fat mass index (BFMI), and fat-free mass index (FFMI). We examined correlations between these indices and clinical measures of COPD severity to evaluate the utility of body composition assessment for early detection of sarcopenia. The study further aimed to validate a clinically feasible, noninvasive method for identifying muscle depletion in stable COPD populations.

## 2 Materials and methods

### 2.1 Study population

This single-center observational study was conducted at the First People’s Hospital of Yinchuan (previously known as the Second Affiliated Hospital of Ningxia Medical University) between January 2023 and January 2024. A total of 237 stable COPD patients and 270 healthy controls were enrolled. All participants were consecutively recruited from the respiratory medicine outpatient and follow-up clinics of the hospital, and baseline clinical data were collected prospectively according to a standardized protocol. The detailed participant recruitment, screening and grouping process is provided in Supplementary Figure S1.

### 2.2 Ethics approval and informed consent

The study protocol was approved by The Clinical Research Ethics Committee of the First People’s Hospital of Yinchuan and adhered to the principles of the Declaration of Helsinki. All participants read and signed the informed consent form as a condition to participate in the study.

The inclusion criteria were as follows: (1) aged ≥ 40 years, (2) stable COPD patients diagnosed according to GOLD guidelines (2023) (13), and willing to participate in this study.

Individuals were excluded from the study if they met any of the following criteria: (1) known history of other major respiratory diseases, such as asthma, active tuberculosis, or lung cancer, (2) presence of systemic inflammatory responses, malignant tumors, connective tissue diseases, or metabolic diseases, (3) a history of psychiatric illness, (4) a contraindication to use body composition analyzer, (5) presence of moderate to severe nutritional risk (NRS-2002 score ≥ 3).

### 2.3 Data collection

#### 2.3.1 Basic demographic data

Age, sex, occupational history, and history of previous illnesses were collected for all participants.

#### 2.3.2 Pulmonary function tests

In this study, the pulmonary function of all subjects was measured by the same physiotherapist using a Japan JESTER pulmonary function instrument (Model: CHESTGRAPH HI-101) in the hospital’s pulmonary function room. Following bronchodilator (salbutamol, 200 μg) inhalation, the forced expiratory volume in the first second/forced vital capacity (FEV_1_/FVC) and forced expiratory volume in 1 s percent predicted value (FEV_1_%pred) were recorded.

Participants were stratified according to GOLD (2023) criteria for FEV_1_%pred (based on FEV_1_/FVC < 0.7).

- Mild COPD (GOLD I): FEV_1_% pred ≥ 80%,
- Moderate COPD (GOLD II): 50% ≤ FEV_1_%pred ≤ 79%,
- Severe COPD (GOLD III): 30% ≤ FEV_1_%pred ≤ 49%,
- Very severe COPD (GOLD IV): FEV_1_%pred < 30%.

#### 2.3.3 Symptom scores

Clinical symptom data were collected via face-to-face interviews by well-trained staff, including the modified Medical Research Council (mMRC) dyspnea score and COPD Assessment Test (CAT).

- Mild symptom group: CAT score (0–10); Mild group: mMRC score (0–1),
- Moderate symptom group: CAT score (11–20); Moderate severe group: mMRC score (2–4).
- Severe symptom group: CAT score (21–30),
- Very severe symptom group: CAT score (31–40).

#### 2.3.4 Acute exacerbation risk assessment

Medical history of COPD patients included history of acute COPD exacerbations, hospitalizations, and routine and/or acute COPD medication use in the past year.

- Low-risk group: 0 or 1 moderate exacerbation and not leading to hospitalization.
- High-risk group: ≥2 moderate exacerbations or ≥1 exacerbation leading to hospitalization.

#### 2.3.5 Body composition test

All body composition measurements were performed by bioelectrical impedance analysis (BIA) using a body composition analyzer (InBody 570, Bysbys Medical Devices Trading Co., Ltd.). Tests were conducted in the morning after an overnight fast, with no strenuous exercise prior to measurement. Subjects stood barefoot on the instrument, made contact with the electrode plates with their feet, grasped the electrode handles with both hands, pressed both thumbs against the electrode points, and kept their arms naturally laterally abducted. The instrument recorded the relevant indicators including body mass index (BMI), fat mass (FM), fat mass index (FMI), fat-free mass (FFM), and fat-free mass index (FFMI).

#### 2.3.6 Diagnostic criteria for concomitant sarcopenia in stable COPD

According to the diagnostic criteria for sarcopenia published by the Asian Working Group for Sarcopenia in 2019 (14): (1) Muscle mass measured by bioelectrical impedance analysis, with SMI as the assessment index, and the judgment criteria: male <7.0 kg/m^2^, female <5.7 kg/m^2^; (2) Muscle strength measured by a handgrip dynamometer, with dominant handgrip strength as the criterion: male < 28 kg, female < 18 kg. Sarcopenia was diagnosed when both criteria (1) and (2) were met.

### 2.4 Statistical analyses

SPSS 27.0, GraphPad Prism 9.5, and OmicStudio software were used to analyze the data and draw graphs. Quantitative data following a normal distribution are expressed as mean ± standard deviation (SD), with intergroup comparisons performed using t-tests and multifactorial analysis of variance. For non-normal distributions, data are presented as median and interquartile range (IQR). Two independent group comparisons were performed using the Mann–Whitney U rank-sum test, and multiple independent group comparisons were performed using the Kruskal-Wallis H test. Categorical data are expressed as cases or percentages (%), with intergroup comparisons made using chi-square tests. Correlation analysis is performed using Pearson’s method or Spearman’s method.

Multiple linear regression were used to explore the relationships between body composition variables and continuous measures of disease severity. Binary logistic regression models were applied to identify independent factors associated with concomitant sarcopenia (binary outcome) in stable COPD patients. Receiver operating characteristic (ROC) curves were plotted to evaluate the clinical application value of body composition indices. The combined diagnostic model of SMI and FEV₁%pred was constructed based on the predicted probability derived from binary logistic regression, and the optimal cut-off value was determined by the maximum Youden index. A *p*-value < 0.05 was considered statistically significant in all analyses.

## 3 Results

### 3.1 Basic clinical characteristics of subjects

In this study, 237 stable COPD patients (166 males and 71 females) and 270 controls (141 males and 129 females) were enrolled. The stable COPD patients group included 40 cases of concomitant sarcopenia (26 males and 14 females). Baseline characteristics of the study groups are presented in Table 1.

**Table 1:** Comparison of clinical data between stable COPD group and healthy control group.

Variable	Stable COPD group (*n* = 237)	Healthy control group (*n* = 270)	t/U/X^2^	*p*-value
Basic clinical data				
Sex			−4.092	<0.001
Male, *n* (%)	166 (70.42)	141 (52.22)		
Female, *n* (%)	71 (29.96)	129 (47.78)		
Age, years	69.00 (64.00, 75.00)	57.00 (49.00, 69.25)	−9.280	<0.001
Height, m	1.68 (1.60, 1.71)	1.65 (1.60, 1.72)	−0.397	0.691
Weight, kg	64.80 (57.45, 75.65)	66.95 (58.58, 78.90)	−1.876	0.061
Body composition index				
BMI, kg/m^2^	24.10 ± 4.02	24.85 ± 3.69	−2.020	0.043
SMI, kg/m^2^	8.32 (6.85, 9.63)	9.94 (8.98, 10.85)	−9.979	<0.001
Male <7.0, *n* (%)	26 (15.66%)			
Female <5.7, *n* (%)	14 (19.72%)			
BFMI, kg/m^2^	7.35 ± 3.07	7.10 ± 2.51	−0.780	0.436
FFMI, kg/m^2^	16.54 (15.43, 18.14)	17.61 (16.04, 19.14)	−4.907	<0.001
Male <17.0, *n* (%)	75 (45.18%)			
Female <15.0, *n* (%)	24 (33.80%)			

Data are presented as means ± SD (quantitative variables) or median and IQR (interquartile range) or *n* (%) (categorical variables). Percentages for sex-specific cut-offs are calculated based on the total number of participants in the corresponding sex group. COPD, Chronic obstructive pulmonary disease; BMI, Body mass index; SMI, Skeletal muscle index; BFMI, Body fat mass index; FFMI, Fat-free mass index.

### 3.2 Comparison of clinical data between stable COPD group and healthy control group

**Figure 1:**
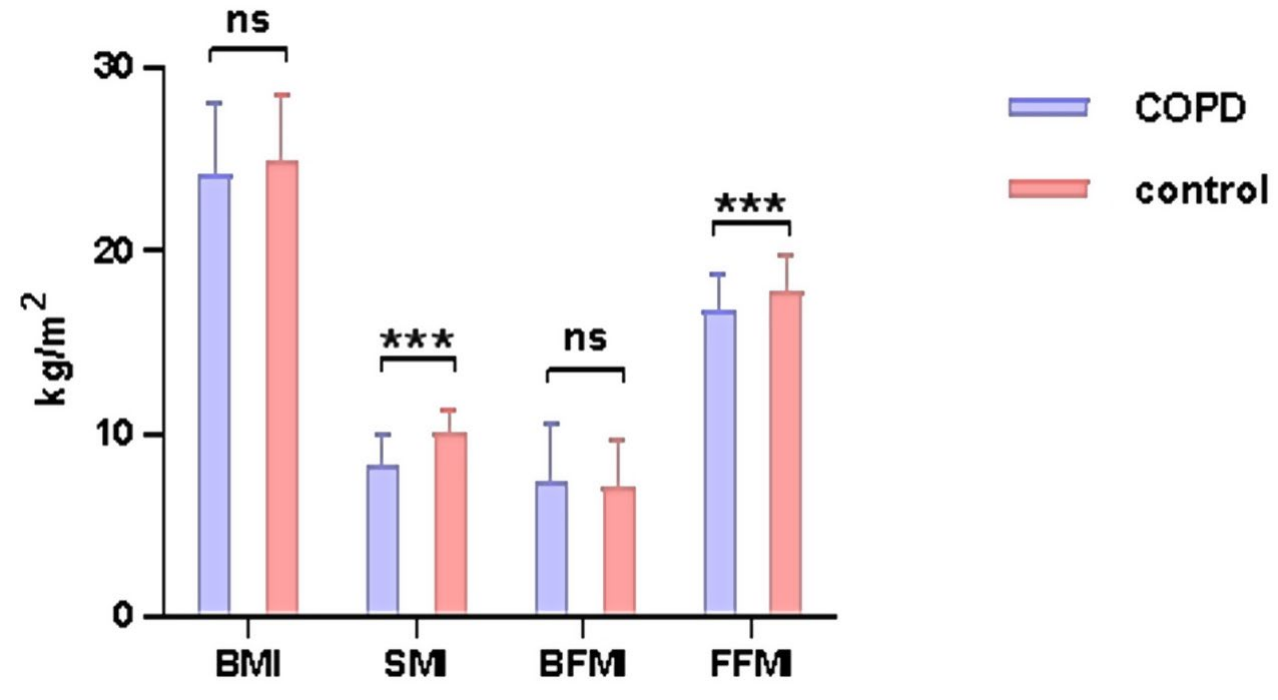
Histogram comparing the variability of body composition between the two groups. The COPD cohort demonstrated significantly reduced BMI, SMI, and FFMI compared to control cohort (*p* < 0.05). * *p* < 0.05, ** *p* < 0.01, *** *p* < 0.001. Error bars represent standard error.

Comparative analyses revealed that stable COPD patients and healthy controls exhibited comparable height, weight, and BFMI. In contrast, the COPD cohort demonstrated significantly reduced BMI, SMI, and FFMI (*p* < 0.05). In the stable COPD group, low skeletal muscle mass was observed in 26 males (15.66% of male patients, SMI < 7.0 kg/m^2^) and 14 females (19.72% of female patients, SMI < 5.7 kg/m^2^). Similarly, low fat-free mass was identified in 75 males (45.18% of male patients, FFMI <17.0 kg/m^2^) and 24 females (33.80% of female patients, FFMI <15.0 kg/m^2^). These findings are presented in Table 1 and Figure 1.

### 3.3 Stratification analysis of stable COPD patients

#### 3.3.1 Stratified by degree of airflow limitation on pulmonary function tests

**Figure 2:**
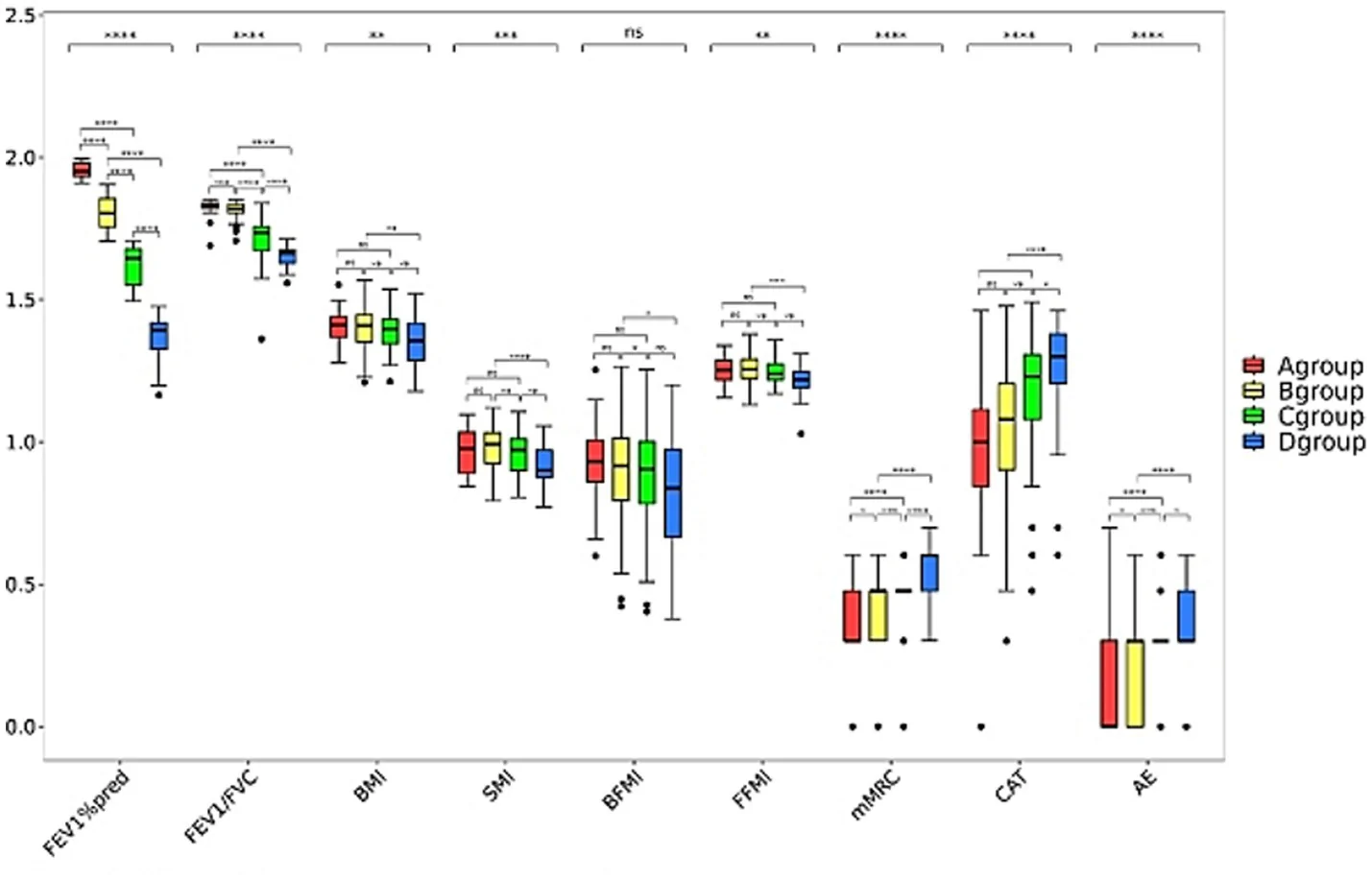
Box plots for analysis of variability of indicators among the four groups. A group: GOLD I, B group: GOLD II, C group: GOLD III, D group: GOLD IV. * *p* < 0.05, ** *p* < 0.01, *** *p* < 0.001.

Significant differences were observed in BMI, SMI, and FFMI (all *p* < 0.05) among COPD patients stratified by GOLD grade. Values were highest in the mild group and lowest in the very severe group, suggesting progressive reductions in these indices correlate with worsening lung function impairment (Table 2, Figure 2).

**Table 2:** Differential analysis of indicators in stable COPD patients with different severity of impaired lung function.

Variable	Mild group (*n* = 51)	Moderate group (*n* = 90)	Severe group (*n* = 60)	Very severe group (*n* = 36)	F/X^2^	*p*-value
Basic clinical data						
Sex					10.265	0.016
Male, *n* (%)	27 (52.94)	64 (71.11)	46 (76.67)	29 (80.56)		
Female, *n* (%)	24 (47.06)	26 (28.89)	14 (23.33)	7 (19.44)		
Age, years	69.02 ± 6.85	69.44 ± 8.40	67.65 ± 8.75	71.22 ± 9.21	3.566	0.312
Height, m	1.64 (1.57,1.70)	1.69 (1.60,1.74)	1.66 (1.61,1.72)	1.70 (1.64,1.71)	5.406	0.144
Weight, kg	66.63 ± 11.32	68.64 ± 12.24	65.16 ± 10.73	60.94 ± 13.29	9.769	0.021
Body composition index						
BMI, kg/m^2^	24.75 ± 3.35	24.72 ± 3.99	23.86 ± 3.75	21.99 ± 4.70	12.427	0.006
SMI, kg/m^2^	8.98 ± 1.79	8.57 ± 1.63	8.26 ± 1.63	7.28 ± 1.50	15.398	0.002
BFMI, kg/m^2^	7.79 ± 2.53	7.75 ± 3.32	7.09 ± 2.97	6.35 ± 3.42	6.411	0.093
FFMI, kg/m^2^	17.05 ± 1.95	16.96 ± 1.84	16.76 ± 1.75	15.54 ± 2.03	14.267	0.003
Symptom scores						
mMRC, scores	1.29 (1.12, 1.47)	1.56 (1.40, 1.71)	1.98 (1.80, 2.16)	2.61 (2.38, 2.84)	66.871	<0.001
CAT, scores	10.08 (8.42, 11.74)	11.67 (10.41, 12.93)	14.78 (12.94, 16.62)	18.25 (16.19, 20.31)	39.912	<0.001
Acute exacerbation risk						
Number of AE hospitalizations in the past year	0.49 (0.27, 0.71)	0.64 (0.52, 0.77)	1.00 (0.83, 1.17)	1.31 (1.08, 1.53)	42.951	<0.001

Data are presented as means ± SD (quantitative variables) or median and IQR (interquartile range) or *n* (%) (categorical variables). BMI, Body mass index; SMI, Skeletal muscle index; BFMI, Body fat mass index; FFMI, Fat-free mass index; mMRC, Modified Medical Research Council; CAT, COPD Assessment Test.

#### 3.3.2 Differential analysis of body composition with different symptoms in stable COPD patients

##### 3.3.2.1 Stratified by mMRC scores

Stable COPD patients were divided into the mild group (*n* = 92) and the moderate–severe group (*n* = 145) according to mMRC scores, as shown in Table 3. Patients in the mild group exhibited significantly higher FEV₁%pred, FEV₁/FVC, SMI, and FFMI, along with significantly lower CAT scores and fewer acute exacerbation hospitalizations compared to the moderate–severe group (all *p* < 0.05). This pattern suggests that greater COPD severity is associated with progressive declines in lung function, reduced skeletal muscle mass, worsening respiratory symptoms, and increased exacerbation frequency.

**Table 3:** Differential analysis of indicators in two groups of stable COPD patients with different mMRC scores.

Variable	Mild group (*n* = 92)	Moderate–severe group (*n* = 145)	t/U/X^2^	*p*-value
Basic clinical data				
Sex			−0.453	0.65
Male, *n* (%)	66 (71.74%)	100 (68.97%)		
Female, *n* (%)	26 (28.26%)	45 (31.03%)		
Age, years	66.42 ± 7.86	70.91 ± 8.19	−4.496	<0.001
Height, m	1.67 ± 0.09	1.65 ± 0.09	−1.898	0.058
Weight, kg	68.77 ± 12.11	64.50 ± 11.77	−2.503	0.012
Pulmonary function				
FEV_1_%pred, %	69.60 (65.56, 73.65)	48.73 (45.17, 52.29)	−6.796	<0.001
FEV_1_/FVC, %	63.04 (61.66, 64.42)	55.25 (53.25, 56.94)	−5.510	<0.001
Body composition index				
BMI, kg/m^2^	24.53 ± 3.59	23.83 ± 4.26	−1.275	0.202
SMI, kg/m^2^	8.90 (8.56, 9.24)	7.91 (7.65, 8.17)	−4.183	<0.001
BFMI, kg/m^2^	7.27 (6.96, 7.84)	7.45 (6.90, 8.00)	−0.358	0.721
FFMI, kg/m^2^	17.33 ± 1.85	16.34 ± 1.92	−3.585	<0.001
Symptom scores				
CAT, scores	7.63 ± 3.837	16.59 ± 5.992	−10.231	<0.001
Acute exacerbation risk				
Number of AE hospitalizations in the past year	0.47 (0.34,0.60)	1.01 (0.90,1.13)	−6.199	<0.001

##### 3.3.2.2 Stratified by CAT scores

Stable COPD patients were divided into the mild group (*n* = 96), the moderate group (*n* = 103) and the severe group (*n* = 38) according to CAT scores, as shown in Table 4. FEV_1_%pred, BMI, SMI, and FFMI demonstrated a progressive decline across severity groups, while mMRC scores and acute exacerbation hospitalization rates increased significantly (*p* < 0.05). This inverse pattern indicates that greater COPD symptom severity is associated with worsening lung function and reductions in skeletal muscle mass.

**Table 4:** Differential analysis of indicators in three groups of stable COPD patients with different CAT scores.

Variable	Mild group (*n* = 96)	Moderate group (*n* = 103)	Severe group (*n* = 38)	F/X^2^	*p*-value
Basic clinical data					
Sex				0.481	0.786
Male, n (%)	68 (70.83)	70 (67.96)	28 (73.68)		
Female, n (%)	28 (29.17)	33 (32.04)	10 (26.32)		
Age, years	67.92 ± 8.34	69.70 ± 7.99	70.89 ± 8.99	6.421	0.040
Height, m	1.67 ± 0.09	1.65 ± 0.09	1.64 ± 0.08	2.885	0.236
Weight, kg	69.50 ± 11.81	65.27 ± 11.57	60.11 ± 11.45	16.295	<0.001
Pulmonary function					
FEV_1_%pred, %	68.70 (64.56, 72.84)	51.50 (47.41, 55.58)	41.32 (34.24, 48.40)	47.692	<0.001
FEV_1_/FVC, %	62.33 (60.82, 63.85)	56.73 (54.80, 58.66)	52.18 (48.65, 55.71)	29.683	<0.001
Body composition index					
BMI, kg/m^2^	24.94 ± 3.83	23.97 ± 3.76	22.37 ± 4.62	9.392	0.009
SMI, kg/m^2^	8.86 ± 1.65	8.14 ± 1.52	7.28 ± 1.53	23.956	<0.001
BFMI, kg/m^2^	7.66 ± 3.03	7.45 ± 3.01	6.46 ± 3.51	3.636	0.162
FFMI, kg/m^2^	17.33 ± 1.84	16.51 ± 1.86	15.81 ± 1.99	17.744	<0.001
Symptom scores					
mMRC, scores	1.16 (1.05, 1.27)	1.96 (1.84, 2.08)	2.79 (2.63, 2.95)	125.488	<0.001
Acute exacerbation risk					
Number of AE hospitalizations in the past year	0.45 (0.32, 0.57)	0.83 (0.72, 0.95)	1.61 (1.38, 1.83)	3.197	<0.001

#### 3.3.3 Stratified by acute exacerbations

According to the number of acute exacerbation hospitalizations in the past year, patients were divided into the low-risk group (*n* = 209) and the high-risk group (*n* = 28), as shown in Table 5. Analysis revealed that the high-risk group had significantly reduced FEV_1_%pred, FEV_1_/FVC ratio, SMI, and FFMI, but significantly elevated mMRC and CAT scores compared to the low-risk group (all *p* < 0.05). This suggests an association between frequent exacerbations and poorer lung function, diminished skeletal muscle mass, and more severe respiratory symptoms.

**Table 5:** Differential analysis of indicators in two groups of stable COPD patients with different risks of acute exacerbations.

Variable	The low-risk group (*n* = 209)	The high-risk group (*n* = 28)	t/U/X^2^	*p*-value
Basic clinical data				
Sex			−0.268	0.789
Male, *n* (%)	147 (70.34)	19 (67.86)		
Female, *n* (%)	62 (29.66)	9 (32.14)		
Age, years	68.95 ± 8.25	70.79 ± 8.98	−1.194	0.232
Height, m	1.66 ± 0.09	1.63 ± 0.08	−1.367	0.172
Weight, kg	66.96 ± 11.79	60.15 ± 12.55	−2.621	0.009
Pulmonary function				
FEV_1_%pred, %	59.08 (56.02, 62.15)	42.90 (34.46, 51.34)	−3.549	<0.001
FEV_1_/FVC, %	59.11 (57.83, 60.40)	51.99 (47.88, 56.11)	−3.289	<0.001
Body composition index				
BMI, kg/m^2^	24.30 ± 3.85	22.64 ± 4.93	−1.603	0.109
SMI, kg/m^2^	8.41 ± 1.62	7.42 ± 1.78	−2.767	0.006
BFMI, kg/m^2^	7.23 (5.21, 9.12)	6.78 (3.59, 10.14)	−0.618	0.537
FFMI, kg/m^2^	16.88 ± 1.87	15.63 ± 2.21	−2.565	0.01
Symptom scores				
mMRC, scores	1.65 ± 0.76	2.64 ± 0.62	−5.913	<0.001
CAT, scores	11.93 ± 6.17	21.93 ± 4.93	−6.794	<0.001

### 3.4 Correlation analysis of body composition indicators and disease assessment indicators in stable COPD patients

**Figure 3:**
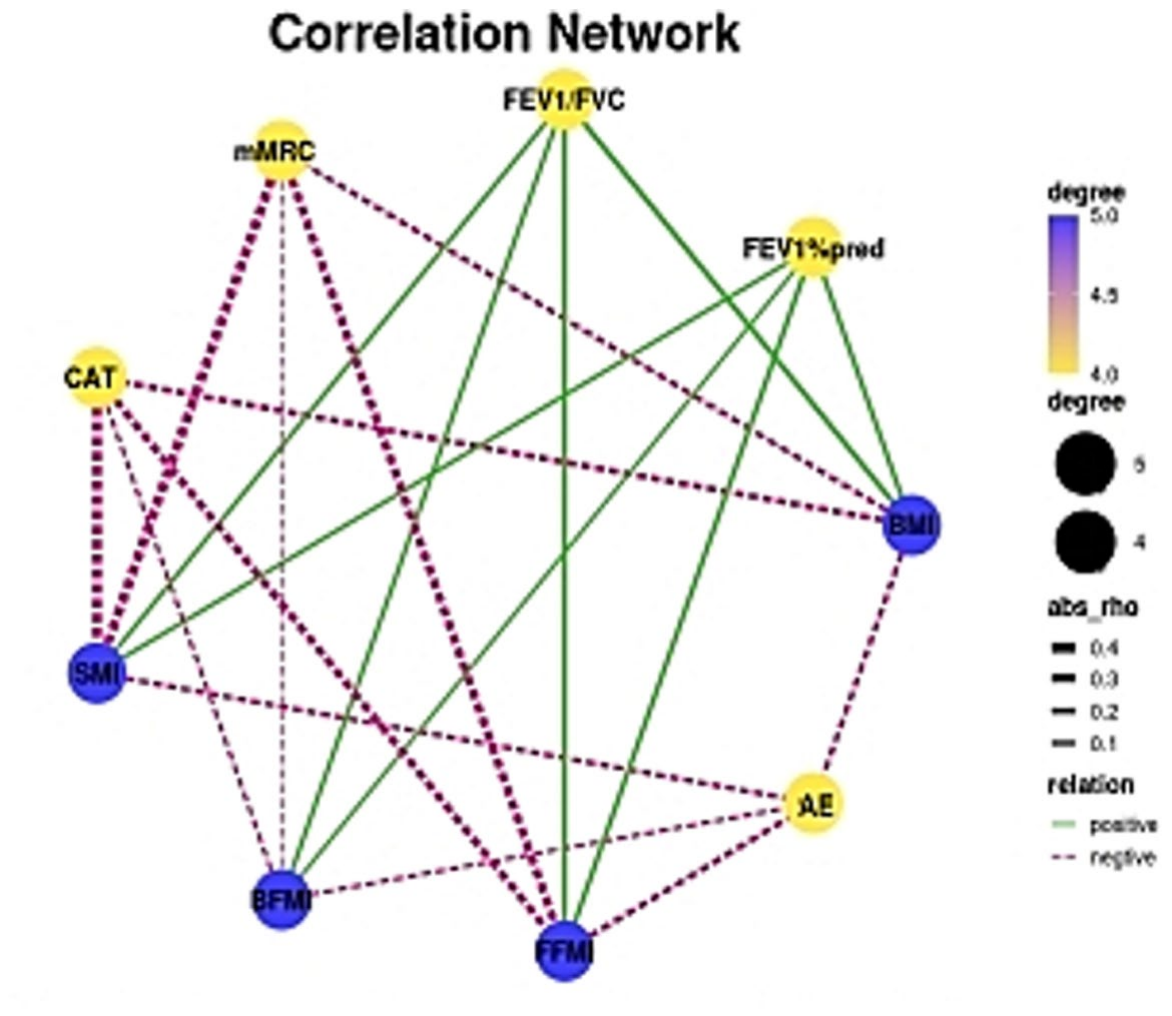
Network diagram for correlation analysis between research indicators.

The statistical results are shown in Table 6 and Figure 3. Further correlation analysis showed that BMI, SMI, and FFMI were positively correlated with FEV_1_%pred and FEV_1_/FVC in the stable COPD group (*r* = 0.142, 0.145, *p* < 0.05; *r* = 0.172, 0.182, *p* < 0.05), and negatively correlated with mMRC score, CAT score, and number of acute exacerbations in the past 1 year (*r* = −0.405, −0.405, −0.174, *p* < 0.01; *r* = −0.336, −0.298, −0.208, *p* < 0.01).

**Table 6:** Correlation analysis of body composition indicators and disease assessment indicators in stable COPD patients.

Variable	BMI		SMI		BFMI		FFMI	
	*r*	*p*	*r*	*p*	*r*	*p*	*r*	*p*
Pulmonary function								
FEV_1_%pred, %	0.162	0.012	0.142	0.029	0.115	0.077	0.172	0.008
FEV_1_/FVC, %	0.194	0.003	0.145	0.025	0.150	0.021	0.182	0.005
Symptom scores								
mMRC, scores	−0.182	0.005	−0.405	<0.001	−0.043	0.515	−0.336	<0.001
CAT, scores	−0.226	<0.001	−0.405	<0.001	−0.119	0.068	−0.298	<0.001
Acute exacerbation risk								
Number of AE hospitalizations in the past year	−0.183	0.005	−0.174	0.007	−0.121	0.063	−0.208	0.001

### 3.5 Binary logistic regression analysis of factors associated with concomitant sarcopenia in stable COPD patients

Table 7 shows that SMI and the FEV_1_%pred were independent predictors of concomitant sarcopenia in stable COPD patients (*p* < 0.05).

**Table 7:** Binary logistic regression analysis of risk factors associated with concomitant sarcopenia in stable COPD patients.

Variable	*OR*	95%*CI*	*P*
FEV_1_%pred	0.868	0.784–0.961	0.006
FEV_1_/FVC	1.066	0.892–1.275	0.481
mMRC scores	0.741	0.108–5.096	0.760
CAT scores	1.076	0.877–1.320	0.482
Acute exacerbation risk	0.910	0.151–5.480	0.918
BMI	2.905	0.014–603.392	0.695
SMI	0.011	0.001–0.090	<0.001
BFMI	0.223	0.001–47.637	0.583
FFMI	0.745	0.004–154.973	0.914

### 3.6 Predictive value of SMI combined with FEV1%pred for early screening of concomitant sarcopenia in stable COPD patients

**Figure 4:**
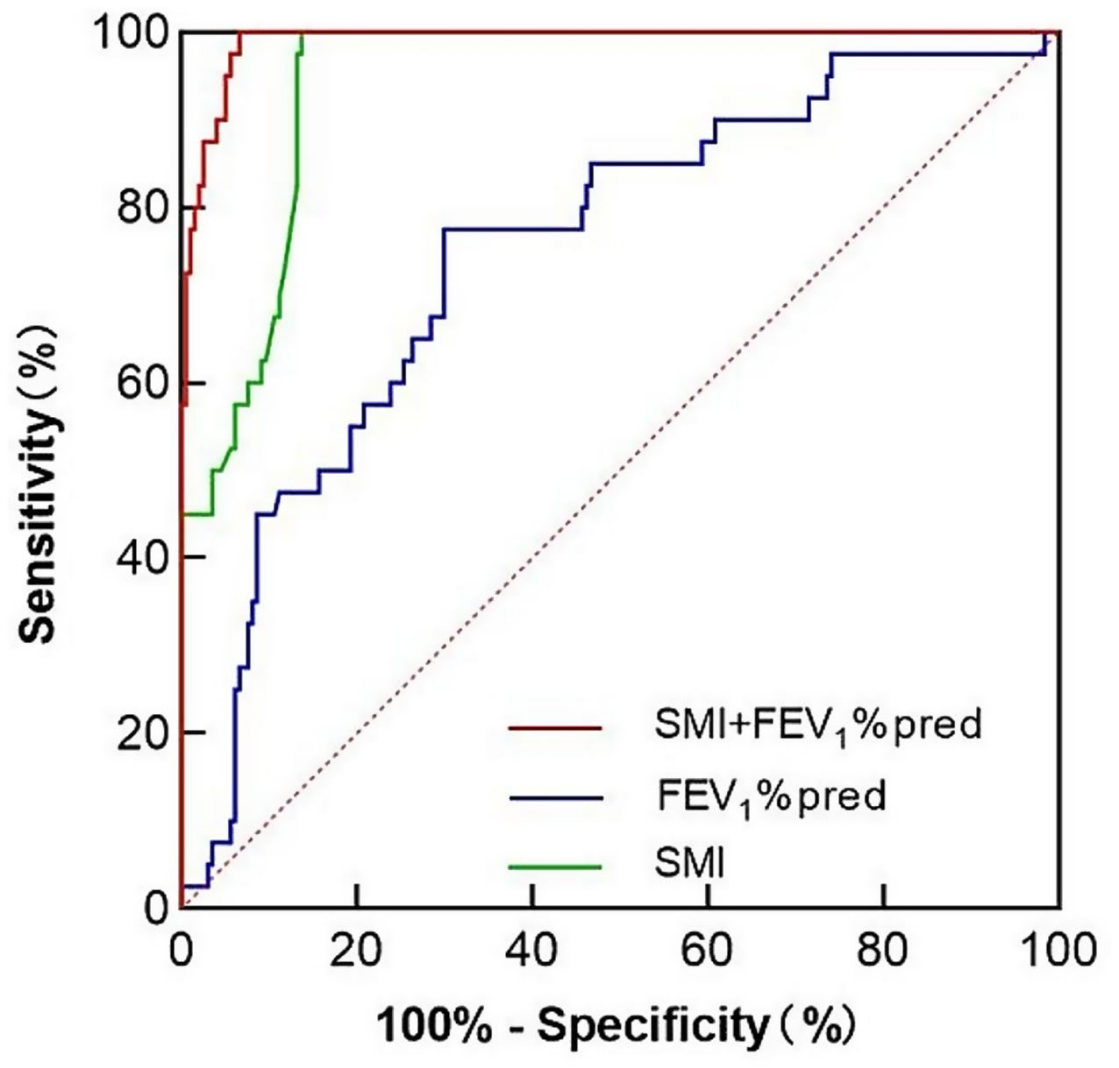
ROC curve analysis of FEV1%pred, SMI, and their combination for predicting concomitant sarcopenia in COPD.

ROC curve analysis based on logistic regression predicted probability revealed that the AUC of SMI combined with FEV₁%pred was 0.990 (95% CI 0.981–0.998). The optimal cut-off value of 0.14 corresponds to the predicted probability of the combined model. The combination of the two indices yielded a larger AUC and a higher sensitivity, suggesting that SMI combined with FEV_1_%pred has favorable predictive value for the early screening of concomitant sarcopenia in stable COPD patients, as shown in Table 8 and Figure 4.

**Table 8:** Predictive efficacy of FEV_1_%pred, SMI, and the combination of the two for concomitant sarcopenia in stable COPD patients.

Variable	AUC	95%CI	*p*-value	Optimal threshold	Specificity %	Sensitivity %	Youden index
FEV_1_%pred	0.750	0.668–0.832	<0.001	49.13	85.00	68.50	0.535
SMI	0.943	0.916–0.971	<0.001	6.99	100.00	86.30	0.863
SMI + FEV_1_%pred	0.990	0.981–0.998	<0.001	0.14	93.40	100.00	0.934

## 4 Discussion

COPD is a chronic inflammatory disease characterized by persistent airflow limitation resulting from airway and/or alveolar damage. Recurrent systemic inflammation extends beyond the respiratory system and contributes to multisystem complications, most notably musculoskeletal impairment (15). As COPD progresses, disrupted homeostasis between skeletal muscle protein synthesis and catabolism leads to progressive muscle loss and functional decline, ultimately driving the development of sarcopenia—a well-recognized extrapulmonary comorbidity of COPD. Its pathogenesis involves multiple pathways including systemic inflammation, oxidative stress, mitochondrial dysfunction, muscle satellite cell injury, and epigenetic regulation (16–20). Previous systematic reviews and epidemiological studies have reported a 20–40% prevalence of sarcopenia in patients with stable COPD, with rates rising alongside disease severity (21, 22). The core manifestations of sarcopenia include reduced muscle mass, decreased strength, and impaired physical function, which in turn reduce exercise tolerance, accelerate functional decline, worsen pulmonary function, and increase the risk of acute exacerbations and hospitalization, ultimately elevating overall morbidity and mortality (23).

In clinically stable COPD, skeletal muscle loss often progresses subclinically without overt symptoms, making it easily overlooked in early stages. Left unaddressed, it may progress to adverse outcomes such as cachexia, dysphagia, falls, and fractures. Routine body composition assessment to detect muscle depletion at an early stage, followed by timely nutritional support and pulmonary rehabilitation, may therefore play an important role in slowing disease progression and improving long-term prognosis (21, 24). In this study, we measured body composition via bioelectrical impedance analysis (InBody 570) in stable COPD patients and healthy controls, and found that SMI and FFMI were significantly lower in the COPD cohort, while BMI did not differ significantly between groups. The clinical implications of these findings are discussed below.

SMI is the core diagnostic indicator for sarcopenia, calculated as appendicular skeletal muscle mass divided by height squared. According to the 2019 consensus update from the Asian Working Group for Sarcopenia, low muscle mass is defined as SMI < 7.0 kg/m^2^ in men and <5.7 kg/m^2^ in women (25). In our cohort, 40 patients (16.88%) had SMI below the threshold, including 11 with mild-to-moderate airflow limitation and 29 with severe-to-very severe disease. This indicates that skeletal muscle depletion occurs across all severity stages of stable COPD, not only in advanced cases. Our finding of reduced SMI in COPD patients is consistent with results from multiple prior studies (13, 14, 24).

FFMI is another key index of lean body mass, calculated as fat-free mass divided by height squared. It directly reflects the relative amount of fat-free tissue (predominantly skeletal muscle) independent of body fat content, offering a more sensitive measure of muscle status than total body weight. Evidence shows that fat-free mass is a protective factor against sarcopenia and an independent predictor of all-cause mortality in COPD patients. Malnutrition in COPD directly drives skeletal muscle atrophy, which is closely linked to functional decline (9, 26). A previous study found that 10–26% of COPD patients experience fat-free mass reduction even without significant overall weight loss (27). In our study, 99 of 237 COPD patients (41.77%) had below-normal FFMI, a rate consistent with prior clinical observations (28). This high prevalence further highlights that muscle depletion is common in stable COPD and often precedes measurable weight loss.

BMI is a widely used indicator of overall nutritional status, reflecting general adiposity. In our study, BMI did not differ significantly between the COPD and control groups, but declined progressively with worsening airflow limitation within the COPD cohort. This pattern can be explained by the relative preservation of fat mass in early-stage COPD, which masks underlying muscle loss—a phenomenon resembling normal-weight sarcopenia (29). While this may appear to echo the ‘obesity paradox’ described in COPD (27), it underscores the fundamental limitation of BMI as a surrogate for muscle mass in this population. Thus, BMI alone is insufficient for detecting early skeletal muscle depletion in stable COPD, especially in patients with mild lung function impairment. Longitudinal BMI monitoring should be complemented by combined assessment of SMI and FFMI to enable timely nutritional intervention.

We further stratified patients by multiple dimensions of disease severity—airflow limitation, symptom burden, and acute exacerbation risk—and found consistent inverse associations: poorer lung function, more severe symptoms, and more frequent exacerbations were all associated with lower SMI and FFMI. This gradient relationship may be mediated by several interrelated factors: chronic hypoxia, persistent systemic inflammation, inadequate nutritional intake, and reduced physical activity collectively suppress protein synthesis and accelerate muscle catabolism, driving progressive muscle wasting. In turn, muscle loss weakens ventilatory mechanics, impairs airway clearance, worsens dyspnea, and further increases exacerbation risk, creating a vicious cycle (7, 30). Our findings extend prior evidence linking sarcopenia to FEV₁ level and GOLD stage (31), by demonstrating associations between body composition indices and both symptom burden and exacerbation frequency. These results support that skeletal muscle status is closely tied to overall disease severity, and that body composition assessment can help identify patients at high risk of poor outcomes.

Several limitations of this study should be considered when interpreting the findings. First, as a single-center cross-sectional study, our design can only demonstrate cross-sectional associations and cannot establish causal relationships between body composition changes and COPD progression. Second, the sample size of patients with sarcopenia was relatively small (n = 40), which may limit the statistical power of subgroup and regression analyses. Third, body composition was measured by BIA, which is convenient for clinical practice but has lower accuracy than the reference standard dual-energy X-ray absorptiometry. Nonetheless, for routine clinical screening and follow-up, the practical advantages of BIA—portability, low cost, and good tolerability—support its use when interpreted alongside clinical context, particularly as a more informative alternative to BMI alone. Fourth, we did not collect detailed data on dietary intake, daily physical activity, or systemic inflammatory markers such as C-reactive protein, which are potential confounding factors for sarcopenia. Additionally, patients with moderate-to-severe nutritional risk were excluded from enrollment, which may have underestimated the overall prevalence of sarcopenia in the stable COPD population and introduced selection bias. Finally, all participants were in the stable phase of COPD, so the findings cannot be directly generalized to patients with acute exacerbations.

Despite these limitations, our findings support the clinical utility of BIA-based body composition assessment in stable COPD follow-up. The combined SMI and FEV₁%pred strategy offers a practical auxiliary screening approach for early sarcopenia identification. Given the underutilization of available treatment approaches for chronic diseases (32), multicenter prospective studies are needed to further validate these findings and clarify causal relationships.

## 5 Conclusion

The combination of SMI and FEV₁%pred has favorable predictive value for early screening of concomitant sarcopenia in stable COPD patients. Therefore, body composition testing may be considered as a supplementary component of routine follow-up for stable COPD patients, alongside pulmonary function testing. It can help clinicians and dietitians perform regular skeletal muscle assessment and nutritional evaluation for stable COPD patients, identify sarcopenia at an early stage, and initiate timely nutritional intervention and pulmonary rehabilitation, so as to improve the overall management of COPD patients.

## Data Availability

The original contributions presented in the study are included in the article/Supplementary material, further inquiries can be directed to the corresponding author/s.

## References

[1] Mou K Chan SMH Vlahos R. Musculoskeletal crosstalk in chronic obstructive pulmonary disease and comorbidities: emerging roles and therapeutic potentials. Pharmacol Ther. (2024) 257:108635. doi: 10.1016/j.pharmthera.2024.108635, 38508342

[2] Wang C Xu J Yang L Xu Y Zhang X Bai C . Prevalence and risk factors of chronic obstructive pulmonary disease in China (the China pulmonary health [CPH] study): a national cross-sectional study. Lancet. (2018) 391:1706–17. doi: 10.1016/S0140-6736(18)30841-9, 29650248

[3] Chen Q Fan Y Huang K Li W Geldsetzer P Bärnighausen T . Cost-effectiveness of population-based screening for chronic obstructive pulmonary disease in China: a simulation modeling study. Lancet Reg Health West Pac. (2024) 46:101065. doi: 10.1016/j.lanwpc.2024.101065, 38721063 PMC11077022

[4] Wang C Qi W Yang T Jiao L Chen Q Huang K . The care cascade of chronic obstructive pulmonary disease in China: a cross-sectional study of individual-level data at enrolment into the national 'happy breathing' Programme. EClinicalMedicine. (2024) 74:102597. doi: 10.1016/j.eclinm.2024.102597, 39114273 PMC11305216

[5] Halpin DMG Dickens AP Skinner D Murray R Singh M Hickman K . Identification of key opportunities for optimising the management of high-risk COPD patients in the UK using the CONQUEST quality standards: an observational longitudinal study. Lancet Reg Health Eur. (2023) 29:100619. doi: 10.1016/j.lanepe.2023.100619, 37131493 PMC10149261

[6] Bhatt SP Abadi E Anzueto A Bodduluri S Casaburi R Castaldi PJ . A multidimensional diagnostic approach for chronic obstructive pulmonary disease. JAMA. (2025) 333:2164–75. doi: 10.1001/jama.2025.7358, 40382791 PMC12086702

[7] Henrot P Dupin I Schilfarth P Esteves P Blervaque L Zysman M . Main pathogenic mechanisms and recent advances in COPD peripheral skeletal muscle wasting. Int J Mol Sci. (2023) 24:6454. doi: 10.3390/ijms24076454, 37047427 PMC10095391

[8] Jiang M Li P Wang Y Cao Y Han X Jiang L . Role of Nrf2 and exercise in alleviating COPD-induced skeletal muscle dysfunction. Ther Adv Respir Dis. (2023) 17:17534666231208633. doi: 10.1177/17534666231208633, 37966017 PMC10652666

[9] Schols AM Ferreira IM Franssen FM Gosker HR Janssens W Muscaritoli M . Nutritional assessment and therapy in COPD: a European Respiratory Society statement. Eur Respir J. (2014) 44:1504–20. doi: 10.1183/09031936.00070914, 25234804

[10] Yang X Wei Y Zhao J Bai Y. Correlation of lung function and sarcopenia in elderly patients with chronic obstructive pulmonary disease and analysis of factors influencing sarcopenia. Am J Transl Res. (2025) 17:358–76. doi: 10.62347/OGUT1532, 39959237 PMC11826181

[11] Wu ZY Lu XM Liu R Han YX Qian HY Zhao Q . Impaired skeletal muscle in patients with stable chronic obstructive pulmonary disease (COPD) compared with non-COPD patients. Int J Chron Obstruct Pulmon Dis. (2023) 18:1525–32. doi: 10.2147/COPD.S396728, 37489239 PMC10363356

[12] Hoz-San Bartolomé P Rodríguez-Hernández C Curbelo YG Ramírez-Fuentes C Vázquez-Ibar O Sanchez-Rodriguez D . The challenge of applying the F-A-C-S pathway from EWGSOP2 for sarcopenia diagnosis in patients with chronic obstructive pulmonary disease: a diagnostic accuracy study. Rehabil. (2025) 59:100879. doi: 10.1016/j.rh.2024.100879, 39985916

[13] Global Strategy for the Diagnosis, Management, and Prevention of Chronic Obstructive Pulmonary Disease. *Global Initiative for Chronic Obstructive Lung Disease (GOLD)*. (2023).

[14] Choi YJ Park HJ Cho JH Byun MK. Low skeletal muscle mass and clinical outcomes in chronic obstructive pulmonary disease. Tuberc Respir Dis (Seoul). (2023) 86:272–83. doi: 10.4046/trd.2023.0008, 37582676 PMC10555524

[15] He Z Wang Y Shan L Mou Y Liu Y Ma G . Reflections on COPD comorbidities. Chin Med J. (2024) 137:1504–6. doi: 10.1097/CM9.0000000000003131, 38750633 PMC11188853

[16] Xu J Zeng Q Li S Su Q Fan H. Inflammation mechanism and research progress of COPD. Front Immunol. (2024) 15:1404615. doi: 10.3389/fimmu.2024.1404615, 39185405 PMC11341368

[17] Liao L Deng M Gao Q Zhang Q Bian Y Wang Z . Predictive and therapeutic value of lipoprotein-associated phospholipaseA2 in sarcopenia in chronic obstructive pulmonary disease. Int J Biol Macromol. (2024) 275:133741. doi: 10.1016/j.ijbiomac.2024.133741, 38986985

[18] Zhang H Qi G Wang K Yang J Shen Y Yang X . Oxidative stress: roles in skeletal muscle atrophy. Biochem Pharmacol. (2023) 214:115664. doi: 10.1016/j.bcp.2023.115664, 37331636

[19] Song Y Han X Wang Y Li K Li H Tian Y . Mitochondrial quality control: a new perspective in skeletal muscle dysfunction of chronic obstructive pulmonary disease. Aging Dis. (2024) 16:3291–310. doi: 10.14336/AD.2024.1129, 39751854 PMC12539542

[20] Attaway AH Bellar A Welch N Sekar J Kumar A Mishra S . Gene polymorphisms associated with heterogeneity and senescence characteristics of sarcopenia in chronic obstructive pulmonary disease. J Cachexia Sarcopenia Muscle. (2023) 14:1083–95. doi: 10.1002/jcsm.13198, 36856146 PMC10067501

[21] Benz E Trajanoska K Lahousse L Schoufour JD Terzikhan N De Roos E . Sarcopenia in COPD: a systematic review and meta-analysis. Eur Respir Rev. (2019) 28:190049. doi: 10.1183/16000617.0049-2019, 31722892 PMC9488535

[22] Kim SH Hong CH Shin MJ Kim KU Park TS Park JY . Prevalence and clinical characteristics of sarcopenia in older adult patients with stable chronic obstructive pulmonary disease: a cross-sectional and follow-up study. BMC Pulm Med. (2024) 24:219. doi: 10.1186/s12890-024-03034-5, 38698380 PMC11067242

[23] Spositon T Oliveira JM Rodrigues A Fonseca J Santin L Machado FVC . Quadriceps weakness associated with mortality in individuals with chronic obstructive pulmonary disease. Ann Phys Rehabil Med. (2022) 65:101587. doi: 10.1016/j.rehab.2021.101587, 34628082

[24] Zhao X Su R Hu R Chen Y Xu X Yuan Y . Sarcopenia index as a predictor of clinical outcomes among older adult patients with acute exacerbation of chronic obstructive pulmonary disease: a cross-sectional study. BMC Geriatr. (2023) 23:89. doi: 10.1186/s12877-023-03784-7, 36774462 PMC9921248

[25] Chen LK Woo J Assantachai P Auyeung TW Chou MY Iijima K . Asian working Group for Sarcopenia: 2019 consensus update on sarcopenia diagnosis and treatment. J Am Med Dir Assoc. (2020) 21:300–307.e2. doi: 10.1016/j.jamda.2019.12.012, 32033882

[26] Cederholm T Barazzoni R Austin P Ballmer P Biolo G Bischoff SC . ESPEN guidelines on definitions and terminology of clinical nutrition. Clin Nutr. (2017) 36:49–64. doi: 10.1016/j.clnu.2016.09.004, 27642056

[27] Van de Bool C Rutten EP Franssen FM Wouters EF Schols AM. Antagonistic implications of sarcopenia and abdominal obesity on physical performance in COPD. Eur Respir J. (2015) 46:336–45. doi: 10.1183/09031936.00197314, 25882802

[28] Shimada T Chubachi S Otake S Sakurai K Sasaki M Iijima H . Differential impacts between fat mass index and fat-free mass index on patients with COPD. Respir Med. (2023) 217:107346. doi: 10.1016/j.rmed.2023.107346, 37390978

[29] Kaluźniak-Szymanowska A Talarska D Tobis S Styszyński A Cofta S Wieczorowska-Tobis K . Body compositions phenotypes of older adults with COPD. Front Nutr. (2024) 11:1449189. doi: 10.3389/fnut.2024.1449189, 39434896 PMC11491889

[30] Wang Y Li P Cao Y Liu C Wang J Wu W. Skeletal muscle mitochondrial dysfunction in chronic obstructive pulmonary disease: underlying mechanisms and physical therapy perspectives. Aging Dis. (2023) 14:33–45. doi: 10.14336/AD.2022.0603, 36818563 PMC9937710

[31] Jin X Yang Y Chen G Shao Y Liu C Li R . Correlation between body composition and disease severity in patients with chronic obstructive pulmonary disease. Front Med (Lausanne). (2024) 11:1304384. doi: 10.3389/fmed.2024.1304384, 38549868 PMC10972851

[32] Lingvay I Cohen RV Roux CWL Sumithran P. Obesity in adults. Lancet. (2024) 404:972–87. doi: 10.1016/S0140-6736(24)01210-8, 39159652

