# Supplementary material for: Body composition in stable COPD: associations with disease severity and early sarcopenia prediction
Source: Front Nutr. 2026 Sep 1;13:1862289. doi: 10.3389/fnut.2026.1862289 (PMC13574661; doi:10.3389/fnut.2026.1862289)
Supplement: Supplementary file 1 [file Table_1.DOCX]

Figure S1. Flowchart of participant recruitment and grouping.

This flowchart illustrates the full recruitment and screening process of the study. All participants were consecutively recruited from the respiratory medicine outpatient clinic, follow-up clinic and health examination center of the First People’s Hospital of Yinchuan between January 2023 and January 2024. Eligible candidates were screened according to pre-specified inclusion and exclusion criteria. A total of 507 participants were finally enrolled in this study, including 237 patients with stable chronic obstructive pulmonary disease (COPD) and 270 age- and sex-matched healthy controls. Patients in the COPD group were further categorized into the sarcopenia subgroup and non-sarcopenia subgroup according to the diagnostic criteria of the Asian Working Group for Sarcopenia (AWGS) 2019.

*Abbreviations*: BIA, bioelectrical impedance analysis; NRS-2002, Nutritional Risk Screening 2002; COPD, chronic obstructive pulmonary disease; AWGS, Asian Working Group for Sarcopenia.
